# Supplementary material for: Potent SARS-CoV-2 neutralizing antibodies with therapeutic effects in two animal models
Source: iScience. 2022 Nov 15;25(12):105596. doi: 10.1016/j.isci.2022.105596 (PMC9664764; doi:10.1016/j.isci.2022.105596)
Supplement: Document S1. Figures S1 and S2 and Tables S1-S5 [file mmc1.pdf]

iScience, Volume *25*

Supplemental information

Potent SARS-CoV-2 neutralizing antibodies with therapeutic effects in two animal models

Masaru Takeshita, Hidehiro Fukuyama, Katsuhiko Kamada, Takehisa Matsumoto, Chieko Makino-Okamura, Tomomi Uchikubo-Kamo, Yuri Tomabechi, Kazuharu Hanada, Saya Moriyama, Yoshimasa Takahashi, Hirohito Ishigaki, Misako Nakayama, Cong Thanh Nguyen, Yoshinori Kitagawa, Yasushi Itoh, Masaki Imai, Tadashi Maemura, Yuri Furusawa, Hiroshi Ueki, Kiyoko Iwatsuki-Horimoto, Mutsumi Ito, Seiya Yamayoshi, Yoshihiro Kawaoka, Mikako Shirouzu, Makoto Ishii, Hideyuki Saya, Yasushi Kondo, Yuko Kaneko, Katsuya Suzuki, Koichi Fukunaga, and Tsutomu Takeuchi

Supplementary Figures

Ab287	-										
Ab326	-	-									
Ab354	-	-	-								
Ab376	-	-	-	-							
Ab496	-	-	-	-	-						
Ab336	-	-	-	-	-	-					
Ab445	-	-	-	-	-	-	-				
Ab159	-	-	-	-	-	-	-	-			
Ab175	-	-	-	-	-	-	-	-	-		
Ab188	-	-	-	-	-	-	-	-	-	-	
Ab308	-	-	-	-	-	-	-	-	-	-	-
	Ab287	Ab326	Ab354	Ab376	Ab496	Ab336	Ab445	Ab159	Ab175	Ab188	Ab308

Figure S1. Epitope binning assay, Related to Figure 3

The results of epitope binning are shown. The minus indicates overlapping epitopes.

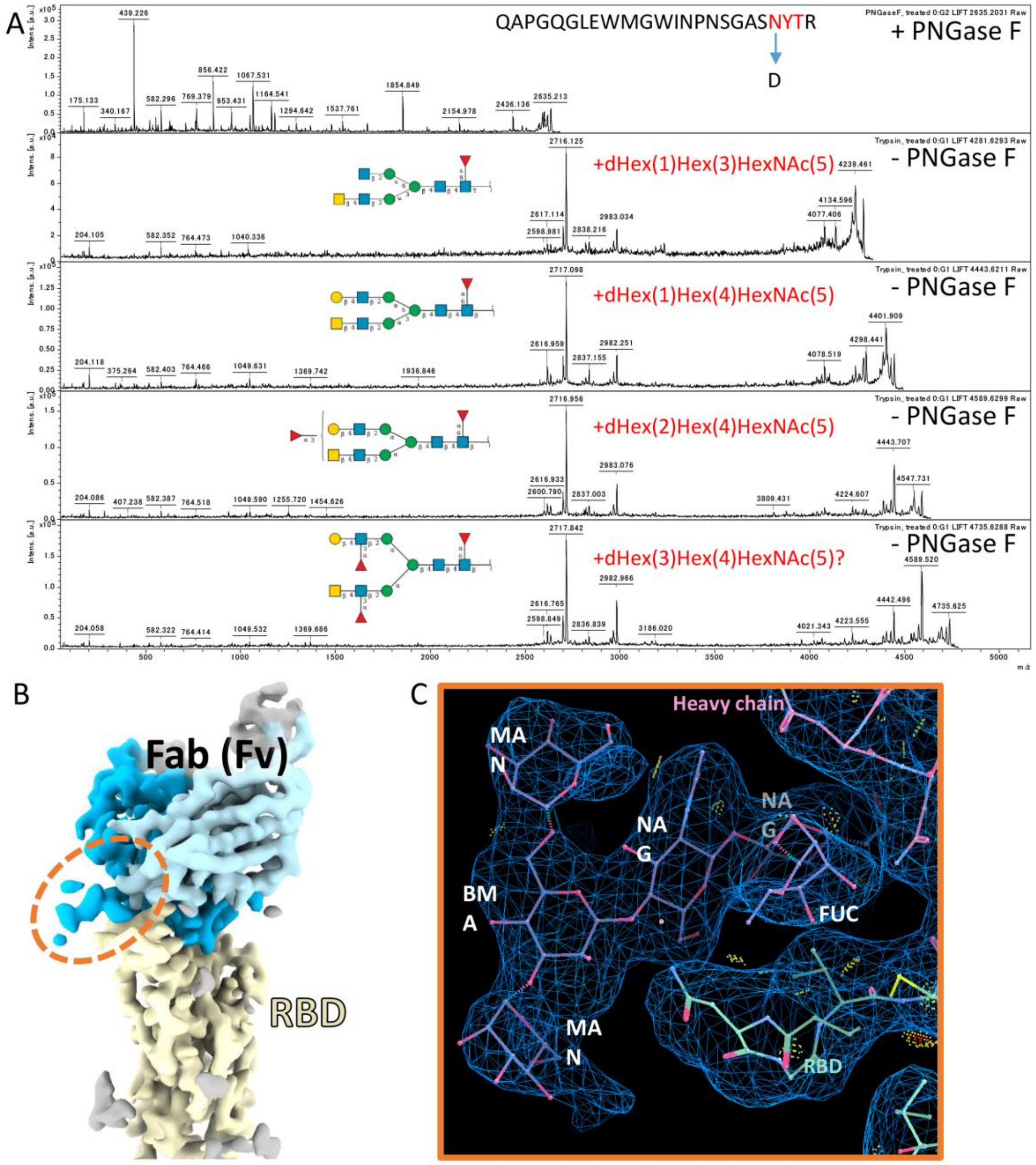

Figure S2. Identification of N-glycan composition, Related to Figure 5

(A) MS/MS spectrograms (300–5000 MW range) of the four candidates that contain a peptide of APGQGLEWMGWINPNSGASNYTR.

(B) Map after local refinement of RBD and the Fab region with specific mask. Dot circle shows the sugar density region of the heavy chain.

(C) The detail density of the sugar moiety with a traceable moiety.

Supplementary Tables

Table S1. The characteristics of patients screened for serum neutralizing ability, Related to Figure 1

	Patients (n = 47)
Sex (male)	25 (52.1)
Age (year)	31.5 (27–55)
Smoking: never / ex / current	35 / 0 / 13
Days between first positive PCR test and first serum collection	48.5 (36.5–58.5)
WHO classification[a]	Critical 2 (4.2)<br>Severe 1 (2.1)<br>Moderate 12 (25.0)<br>Mild 17 (35.4)<br>Asymptomatic 16 (33.3)
<Signs and symptoms>	
Fever (≥37.5°)	26 (54.2)
Upper respiratory symptoms[b]	24 (50.0)
Lower respiratory symptoms[c]	18 (37.5)
Pneumonia	15 (31.3)
<Laboratory data>	
Lowest lymphocyte count (/μl)	1370 (967–1915)
Highest LD level (U/l)	190 (158–235)
Highest CRP level (mg/dl)	0.25 (0.03–1.3)
<Treatment>	
Systemic corticosteroids	4 (8.3)

Data are shown as numbers, numbers (%), or medians (interquartile ranges), as appropriate. [a]Disease severity is based on "Living guidance for clinical management of COVID-19," edited by the WHO (23 Nov 2021). [b]Upper respiratory symptoms include rhinorrhea, sore throat, loss of smell, and loss of taste. [c]Lower respiratory symptoms include cough and sputum production.

WHO, World Health Organization; PCR, polymerase chain reaction; LD, lactate dehydrogenase; CRP, C-reactive protein

Table S2. The characteristics of patients for antibody production, Related to Figure 2

Sample ID	Sex	Age	Days after first positive PCR	Severity	Fever	Pneumonia	Systemic steroid use
COV003	male	60	29	severe	+	+	+
COV003-2	male	60	103				
COV011	male	65	39	critical	+	+	+
COV011-2	male	65	60				
COV017	female	20	10	moderate	+	+	-
COV019	female	30	55	mild	+	-	-
COV025	female	36	44	moderate	+	+	-
COV027	female	35	49	mild	+	-	-
COV047	male	44	46	mild	+	-	-
COV052	male	29	28	moderate	+	+	-
COV080	male	33	62	moderate	+	+	-
COV081	male	59	69	moderate	+	+	-
COV082	female	65	53	moderate	+	+	-
COV088	male	52	56	critical	+	+	+

PCR, polymerase chain reaction

Table S3. Cryo-EM data collection, refinement, and validation statistics for Spike RBD/Fab complexes, Related Figure to 5

complexed with	Ab159	Ab188	Ab326	Ab354	Ab445	Ab496 (Fv-clasp)
EMD ID	33060	33061	33062	33059	33064	33063
PDB ID	7X8Y	7X8Z	7X90	7X8W	7X92	7X91
**Data collection and processing**						
Voltage (kV)	300	300	300	300	300	300
Electron exposure ($e^-/Å^2$)	50	50	50	50	50	50
Defocus range (μm)			-0.8 to -2.4			
Pixel size (Å)	0.829	0.829	0.829	0.829	0.829	0.829
Symmetry imposed	*C*1	*C*1	*C*1	*C*1	*C*1	*C*1
Particles in final reconstruction (no.)	155607	69153	42808	74848	64299	79377
Map resolution (Å) postprocess	4.1	4.1	4.2	3.1	4.1	4.3
FSC threshold	0.143	0.143	0.143	0.143	0.143	0.143
Map sharpening B factor ($Å^2$)	-198	-178	-171	-118	-180	-199
**Refinement**						
Initial model used	7X8W	7X8W	7X8W	5CCK	7X8W	7X8W
RBD mode used	up	up	down	down	up	up
Model resolution (Masked) (Å)	4.3	4.3	4.3	3.3	4.3	4.4
FSC threshold	0.5	0.5	0.5	0.5	0.5	0.5
**Model composition**						
Non-hydrogen atoms	3435	3333	3301	3460	3358	3363
Protein residues	440	426	420	435	426	427
Ligands	1	1	2	8	2	2
B factors ($Å^2$)						
Protein	111.02	85.93	91	103	97	132
Ligand	88.78	96.08	108	128	114	130
R.m.s. deviations						
Bond lengths (Å)	0.002	0.003	0.003	0.003	0.005	0.003
Bond angles (°)	0.522	0.578	0.568	0.496	0.734	0.615
**Validation**						
MolProbity score	1.89	2.16	2.11	1.61	2.26	2.13
Clashscore	6.67	10.46	9.9	4.44	12.5	11.08
Poor rotamers (%)	0	0	0	0	0.27	0
Ramachandran plot						
Favored (%)	91.01	87.14	88.16	94.17	85.48	89.31
Allowed (%)	8.99	12.62	11.84	5.83	14.52	10.69
Disallowed (%)	0	0.24	0	0	0	0

Table S4. Primer sequences, Related to Figure 2

		5' - 3' sequence
primer set1	Forward	CAGTGGTATCAACGCAGAGTACG
	Reverse IgG	TGAGTTCCACGACACCGTCAC
	Reverse IgA	TTCGCTCCAGGTCACACTGAG
	Reverse IgM	ACAAAGTGATGGAGTCGGGAAG
	Reverse Igκ	TCCGAGCTCGGTACCAAGCTAACACTCTCCCCTGTTGAAGCTC
	Reverse Igλ	TCCGAGCTCGGTACCAAGCTATGAACATTCTGTAGGGGCCACT
		TCCGAGCTCGGTACCAAGCTATGAACATTCCGTAGGGGCAAC
		TCCGAGCTCGGTACCAAGCTATGAACATTCTGCAGGGGCC
primer set2	Forward IgH	TGAGCTACGGACTCGAGCAGGTGCAGCTGGTGCAG
		TGAGCTACGGACTCGAGGAGGTGCAGCTGGTGCAG
		TGAGCTACGGACTCGAGGAGGTGCAGCTGGTGGAG
		TGAGCTACGGACTCGAGGAGGTGCAGCTGTTGGAG
		TGAGCTACGGACTCGAGCAGGTGCAGCTGCAGGAG
		TGAGCTACGGACTCGAGCAGGTGCAGCTACAGCAGTG
		TGAGCTACGGACTCGAGCAGGTTCAGCTGGTGCAG
		TGAGCTACGGACTCGAGCAGGTCCAGCTGGTACAG
		TGAGCTACGGACTCGAGCAGGTGCAGCTGGTGGAG
		TGAGCTACGGACTCGAGGAAGTGCAGCTGGTGGAG
		TGAGCTACGGACTCGAGCAGCTGCAGCTGCAGGAG
		TGAGCTACGGACTCGAGCAGGTACAGCTGCAGCAG
	Forward Igκ	TGAGCTACGGACTCGAGGACATCCAGATGACCCAGTC
		TGAGCTACGGACTCGAGGACATCCAGTTGACCCAGTCT
		TGAGCTACGGACTCGAGGCCATCCGGATGACCCA
		TGAGCTACGGACTCGAGGATATTGTGATGACCCAGACTCC
		TGAGCTACGGACTCGAGGATATTGTGATGACTCAGTCTCC
		TGAGCTACGGACTCGAGGATGTTGTGATGACTCAGTCTCC
		TGAGCTACGGACTCGAGGAAATTGTGTTGACACAGTCTCC
		TGAGCTACGGACTCGAGGAAATAGTGATGACGCAGTCTCC
		TGAGCTACGGACTCGAGGAAATTGTGTTGACGCAGTCT
		TGAGCTACGGACTCGAGGACATCGTGATGACCCAGTC
	Reverse Igλ	TGAGCTACGGACTCGAGCAGTCTGTGCTGACKCAGC
		TGAGCTACGGACTCGAGCAGTCTGCCCTGACTCAGC
		TGAGCTACGGACTCGAGTCCTATGAGCTGACWCAGCC
		TGAGCTACGGACTCGAGCAGCYTGTGCTGACTCAGTC

		TGAGCTACGGACTCGAGAATTTTATGCTGACTCAGCCG<br>TGAGCTACGGACTCGAGCAGRCTGTGGTGACYCAG
	Reverse IgG	GATGGGCCCTTGGTGGA
	Reverse IgA	TCACACTGAGTGGCTCCTGG
	Reverse IgM	TCGTATCCGACGGGGAATTC
	Reverse Igκ	GGAAGATGAAGACAGATGGTG
	Reverse Igλ	AGCTCCTCAGAGGAGGGC<br>TCCTCAGAGGAGGGTGG
primer set3	Forward IgH	ACAGGTGCCCACTCCCAGGTGCAG<br>AAGGTGTCCAGTGTGARGTGCAG<br>CCCAGATGGGTCCTGTCCCAGGTGCAG<br>CAAGGAGTCTGTTCCGAGGTGCAG
	Forward Igκ	ATGAGGSTCCCYGCTCAGCTGCTGG<br>CTCTTCCTCCTGCTACTCTGGCTCCCAG<br>ATTTCTCTGTTGCTCTGGATCTCTG
	Reverse Igλ	GGTCCTGGGCCCAGTCTGTGCTG<br>GGTCCTGGGCCCAGTCTGCCCTG<br>GCTCTGTGACCTCCTATGAGCTG<br>GGTCTCTCTCSCAGCYTGTGCTG<br>GTTCTTGGGCCAATTTTATGCTG<br>GGTCCAATTCYCAGGCTGTGGTG<br>GAGTGGATTCTCAGACTGTGGTG
	Reverse IgG	GATGGGCCCTTGGTGGA
	Reverse IgA	TCACACTGAGTGGCTCCTGG
	Reverse IgM	TCGTATCCGACGGGGAATTC
	Reverse Igκ	GGAAGATGAAGACAGATGGTG
	Reverse Igλ	AGCTCCTCAGAGGAGGGC<br>TCCTCAGAGGAGGGTGG
primer set4	Forward	Optimized for each cell
	Reverse IgG	GATGGGCCCTTGGTGGA
	Reverse IgA	Optimized for each cell
	Reverse IgM	Optimized for each cell
	Reverse Igκ	GGAAGATGAAGACAGATGGTG
	Reverse Igλ	AGCTCCTCAGAGGAGGGC<br>TCCTCAGAGGAGGGTGG

Table S5. The sequences of selected antibodies, Related to Figure 4 and Figure 5

Ab159 Heavy chain

CAGGTGCAGCTGGTGGAGTCTGGGGGAGGCTTGGTAAAGCCTGGGGGGTCCCTTAGACTCTCCTG
TGCAGCCTCTGGATTCACTTTCAATAACGCCTGGATGAGCTGGGTCCGCCAGGCTCCAGGGAAGG
GGCTGGAGTGGGTTGGCCGTATTAAAAGCAAAACTGATGGTGGGACAACAGACTACGCTGCACCC
GTGAAAGGCAGATTCACCTTCTCAAGAGATGATTCAAAAAACACGCTGTATCTGCAAATGAGCAGCC
TGAAAACCGAGGACACAGCCCTGTATTACTGTACCACAGCGGATTACGATATCTTGACTGGTACCC
CCGCCTCCCCATACTGGGGCCAGGGAACCCTGGTCACCGTCTCCTCAG

Ab159 Light chain (Kappa)

GACATCCAGATGACCCAGTCTCCATCCTCCCTGTCTGCATCTGTAGGAGACAGAGTCACCATCACTT
GCCGGGCAAGTCAGAGCATTACCAACTATTTAAATTGGTATCAGCAGAAACCAGGGAAAGCCCCTA
AATTCCTGATCTATGCTGCATCCAGTTTGCAAGGTGGGGTCCCATCAAGGTTCCGTGGCAGTGGAT
CTGGGACAGATTTCACTCTCACCATCAGCAGTCTGCAACCTGAAGATTTTGCAACTTACTACTGTCA
ACAGACTTACAGTACCCTCTGGACGTTCGGCCAAGGGACCAAGGTGGAAATCAAAC

Ab188 Heavy chain

GAAGTGCAGCTGGTGGAGTCTGGGGGAGGCTTGGTACAGCCTGGGGGGTCCCTGAGACTCTCCT
GTGCAGCCTCTGGATTCACCTTCATTAACTATAAAATGAACTGGGTCCGCCAGGCTCCAGGGAAGG
GGCTGGAGTGGGTTTCATACATTAGTAGTGGTAGTGATGCCATTTACTACGCAGACTCTGTGAAGG
GCCGATTCACCATCTCCAGAGACAATGCCAAGAACTCACTGTATCTGCAAATGAACAGCCTGAGAG
ACGAGGACACGGCTCTGTATTACTGTGCGAGAGGGGGTGGATACAACTATGGCGAGTGTATGGAC
GTCTGGGGCCAAGGGACCACGGTCACCGTCTCCTCAG

Ab188 Light chain (Kappa)

GACATCCAGATGACCCAGTCTCCAGACTCCCTGGCTGTGTCTCTGGGCGAGAGGGCCACCATCAA
CTGCAAGTCCAGCCAGAGTGTTTTATACAGCTCCAACAATAAGAACTACTTAGCTTGGTACCAGCAG
AAACCAGGACAGCCTCCTAAACTGCTCATTTACTGGGCATCTACCCGGGAATCCGGGGTCCCTGAC
CGATTCAGTGGCAGCGGGTCTGGGACAGATTTCACTCTCACCATCAGCAGCCTGCAGGCTGAAGAT
GTGGCAGTTTATTACTGTCAGCATTATTATAGTCCTCCTCCCACTTTCGGCGGAGGGACCAAGGTG
GAGATCAAAC

Ab326 Heavy chain

GAAGTGCAGCTGGTGGAGTCTGGGGGAGGCGTGGTCCAGCCTGGGAGGTCCCTGAGACTCTCCT
GTGCAGCCTCTGGATTCACCTTCAGTCACTATCCTATGCACTGGGTCCGCCAGGCTCCAGGCAGGG
GGCTGGAGTGGGTGGCAGTTATATCATATGATGGAAGTAATCAACACTACGTAGACTCCGTGAAGG
GCCGATTCACCATCTCCAGAGACAATTCCAAGAACACGCTGTATTTGCAAATGAACAGCCTGAGAG
CTGAGGACACGGCTGTGTATTTCTGTGCGAGCCTAATTACTATGATTCGGGGAGTTCCCGACTACT
GGGGCCAGGGAACCCTGGTCACCGTCTCCTCAG

Ab326 Light chain (Kappa)

GACATCCAGATGACCCAGTCTCCATCCTCCCTGTCTGCATCTGTTGGAGACAGAGTCACCATCACTT
GCCGGGCAAGTCAGAGCATTAGCAACTATTTAAATTGGTATCAGCAGAAACCAGGGAAAGCCCCTA

ACCTCCTAATCTTTGCTGCATCCAGTTTGCAAAGTGGGGTCCCATCAAGGTTCAGTGGCAGTGGAT
CTGGGACAGAATTCACTCTCACCATCAGCAGTCTGCAACCTGAAGATTTTGCAGCTTACTACTGTCT
GCAGACTTACAGTACCCCTCGTACTTTTGGCCAGGGGACCAGGCTGGAGATCAAAC

Ab354 Heavy chain

CAGGTGCAGCTGGTGCAGTCTGGGGCTGAGGTGAAGAAGCCTGGGGCCTCAGTGACGGTCTCCT
GCAAGGCTTCTGGATTCCTCTTCACCGGCTACTATATGCACTGGGTGCGACAGGCCCCTGGACAAG
GGCTTGAGTGGATGGGATGGATCAACCCTAACAGTGGTGCCTCAAACTATACACGGAAATTTCAGG
GCAGGGTCACCATGACCAGGGACACGTCCATCAGCGCTACCTACATGAAACTGAGCAGGCTGACA
TCTGACGACACGGCCGTGTATTACTGTGCGAGAGATCTCGCTTTTAGTATGGTTCGGGGAGCTCTT
GACTACTGGGGCCAGGGAACCCTGGTCACCGTCTCCTCAG

Ab354 Light chain (Lambda chain)

CAGCTTGTGCTGACTCAGCCTCCCTCCGCGTCCGGGTCTCCTGGACAGTCAGTCACCATCTCCTGC
ACTGGAACCAGCAGTGACGTTGGTGGTTATAACTATGTCTCCTGGTACCAACATCACCCAGGCAAA
GCCCCCAAACTCATGATTTATGAGGTCAGTAAGCGGCCCTCAGGGGTCCCTGATCGCTTCTCTGGC
TCCAAGTCTGGCAACACGGCCTCCCTGACCGTCTCTGGGCTCCAGGCTGAAGATGAGGCTGATTAT
TACTGCAACTCATATGCAGGCAACAACAATTGGGTCTTCGGAACTGGGACCAAGGTCACCGTCCTA
G

Ab445 Heavy chain

GAAGTGCAGCTGGTGGAGTCTGGGGGAGGCGTGGTCCAGCCTGGGACGTCCCCGAGACTCTCCT
GTGCAGCGTCTGGATTCACCTTCAGTAACTCTGGCATGCACTGGGTCCGCCAGGCTCCAGGCAAG
GGGCTGGAGTGGGTGGCAGTTATATGGTATGATGGAAGTAAAAAATATTATGTAGACTCCGTGAAG
GGCCGATTCACCATCTCCAGAGACAATTCCAAGAACACGCTGTATCTGCAAATGAACAGCCTGAGA
GCCGAGGACACGGCTGTGTATTACTGTGCGAGAGATGGGACCGTAGCAGTTCGGGGAGTTATGAA
CCCGTTTTTTGACTACTGGGGCCAGGGAACCCTGGTCACCGTCTCCTCAG

Ab445 Light chain (Kappa)

GACATCCAGTTGACCCAGTCTCCATCCTCCCTGTCTGCATCTGTAGGAGACAGAGTCACCATCACTT
GCCAGGCGAGTCAGGACATTAGCAACTATTTAAATTGGTATCAGCAGATACCAGGGAAAGCCCCTA
AGCTCCTGATCTACGATGCATCCAATTTGGAAACAGGGGTCCCATCAAGGTTCAGTGGAAGTGGAT
CTGGGACAGATTTTACTTTCACCATCAGCAGCCTGCAGCCTGAAGATATTGCAACATATTACTGTCA
ACAGTATGATAATCTCCCGTACACTTTTGGCCAGGGGACCAAGCTGGAGATCAAAC

Ab496 Heavy chain

GAGGTGCAGCTGGTGCAGTCTGGGCCTGAGGTGAAGAAGCCTGGGACCTCAGTGAAGGTCTCCTG
CAAGGCTTCTGGATTCATCTTTACTAACTCTGCTGTGCAGTGGGTGCGACAGGCTCGTGGACAACG
CCTTGAGTGGATAGGATGGATCGTCGTTGGCAGTGGTAACCCAAACTACGCACAGAAGTTCCAGGA
AAGAGTCACCATTACCAGGGACAGGTCCACAAGCACAGCCTACATGGAGCTGAGCAGCCTGACATC
CGAGGACACGGCCGTGTACTACTGTGCGGCCTTCCCCTATTACTATGATAATAGTGGCTCCGGGCC
GTGGGGCCAGGGAACCCTGGTCACCGTCTCCTCAG

Ab496 Light chain (Kappa)

GACATCCAGTTGACCCAGTCTCCATCCTCCCTGTCTGCATCTGTAGGAGACAGAGTCACCATCACTT
GCCAGGCGAGTCAGGACATTAGGAACAATTTAAATTGGTATCAGCAGATACCAGGGAAAGCCCCTA
AGCTCCTGATCTACGATGCATCCAATTTGGAAACAGGGGTCCCATCAAGGTTCAGTGGAAGTGCAT
CTGGGACAGATTTTACTTTCACCATCAGCAGTCTGCAGCCTGAAGATGTTGCAACATATTACTGTCA
ACAGTATGCTAATCTCCCTCCATTCACTTTCGGCCCTGGGACCAAAGTGGATATCAAAC
